# The Cyprus Society

**Published:** 1890-02-01

**Authors:** 


### The Cyprus Society.

### I.—HOSPITAL WORK.

We should have been glad to have found an earlier opportunity of alluding to the work which this society has taken in hand, and which we hope it will succeed in accomplishing. The society has been formed to promote hospital and educational work in Cyprus, and to advance any and all other measures of a philanthropic kind in that island which may win the approval of the members, and which the society may possess the means of assisting. The hospital and educational departments are the primary and pressing points in its programme, and any of the needed help which can be given to the Cypriots under these heads will be offered to our fellow-subjects without distinction of race or creed, and without any motives of proselytism.

Politicians may go on discussing the advisability or the reverse of the acquisition of Cyprus; statesmen have the higher task of overcoming the obstacles to good government which Cyprus and the Cypriots present for solution; but philanthropists have a splendid field for their highest efforts, impeded by nothing but the labour of showing what ought to be done, and collecting the money to do it. These latter are real labours, to be sure; the diffusion of information and the getting in of subscriptions are things which call for real toil and untiring zeal. But the workers have everything in their favour. "Cyprus has claims upon us," is about as much as most people here at home know of the place at present. A great many would be grateful for information about our government and our fellow-subjects there. "Hospital and educational work" is both a practical and a popular programme. Let it be known that we wish to extend to others some of our own supreme advantages in these matters—to others almost wholly without them, but most willing to receive them—and surely many hands will be stretched out to aid and push forward such an admirable enterprise.

Cyprus is about 100 miles in length, with an average breadth of fifty miles. The area is about 3,584 square miles. There was a population in 1881 of 186,173 persons. About one-fourth of these are Mahometans; nearly all the rest belong to the Orthodox Greek Church, though Maronites, members of the Church of England, Roman Catholics, Presbyterians, and Wesleyans are also counted amongst the population.

The whole island is marked out into six districts, and a word might be said of the hospital accommodation, or the absence thereof, in these various districts. In the district which includes the capital, Nicosia, there is a small hospital maintained by Government, and a new hospital in this district (in the first instance for ophthalmic diseases) is projected as a memorial of the Queen's Jubilee. In three other districts—Larnaca, Limassol, and Papho—there are small hospitals, but "owing to the want of funds," says the Blue-book, "the hospital at Papho has for some time had almost entirely to suspend its operations, and, notwithstanding the energetic endeavours of the commissioner in that district to collect support, no satisfactory result has been arrived at." From an unofficial source we learn that "in the Famagusta district a hospital was opened a few years ago in a hired house, but for want of funds it had to be closed." This accounts for five of the districts, and the story of the South is briefly told: "In the district of Kyrenia there has never been a hospital."

The Cyprus Society thinks that if it had been born sooner it could, by a timely grant, have saved the Papho Hospital, but is confident, at all events, that its duty now is to build a hospital at Kyrenia.

The chief medical officer's figures of a year's work may not be without interest. Dr. F. C. Heidenstain says: "At the Nicosia Hospital, 77 were treated; of these, 55 were discharged cured, 11 relieved, 6 died, 5 remaining under treatment. At the Larnaca Hospital, 112 were treated; of these 57 were discharged cured, 31 relieved, 5 not improved, 16 died, 3 remaining under treatment. At the Limassol Hospital 224 patients were treated; of these 208 were discharged cured, 2 relieved, 3 not improved, and 11 died." Dr. Heidenstain offers a criticism on these figures. He says: "The rather high death-rate in the hospitals can only be accounted for, as I have mentioned on other occasions, by the fact that patients generally, especially those from the district, are brought in when their cases are almost hopeless." It is a remark which one sometimes hears from a doctor in private as well as in hospital practice. But may not the matter of hospital equipment, the qualifications of nurses, and such things, have some direct bearing on these results with which the chief medical officer is so ill satisfied? And, if so, is it not a pity to say so plainly? We are accustomed to consider a Blue-book a kind of collection of grim realities; but, according to what one hears of Cyprus hospitals from other sources, it is a comparatively cheerful picture, in this case, which the Blue-book presents. Are not trained nurses something like unknown in the island of Cyprus? Are not the hospitals sometimes mere houses where beds and food are provided, with such medical attendance as a man can give who is much about in his district? Are one or two native servants able to keep a hospital in order in a hot climate, with ignorance and dirt reigning supreme? Have not many women and children to be refused admission, whose cases might be met by proper nursing, food, and even a little attention from the doctor in charge? Everything, it is true, cannot be done in a day; and everything, perhaps, cannot and ought not to be done by the Government. The Government has done something, and is doing more; but there is evidently room for all that this Cyprus Society can accomplish, a good use to be made of every shilling it can collect from the charitably disposed.

The society has decided that the first and earliest attention must be given to the nursing department of hospital work in Cyprus. It is considered that for £180 a year two trained nurses could be sent out, and a real beginning made in the matter of training native servants as nurses. It will certainly be possible to raise this sum for such a purpose. Selecting really suitable women for this mission will be more difficult. It is a splendid opening for good work to those who would value it and could sustain the labour it would entail. It is essentially a case where one should be a nurse first and a lady afterwards, though it has been added—truly or not—that only ladies should volunteer for this service, as no one else could sustain the monotony of life in the island. To ourselves, it looks as if the work itself would banish any thought of monotony. We hope, however, to hear soon that the suitable nurses have been found, and are on their way to Cyprus.

Another project is that of developing a system of "locomotive dispensaries" and ambulances, and offering the inhabitants of every part of the island the relief and treatment which can be thus afforded. The Cyprus Society is likely to achieve this as well as the other undertakings it has in hand, for its members go to work with a competent knowledge of existing needs, and in full sympathy with the feelings of the Cypriots themselves. Men take a prominent share in its counsels, who have resided in Cyprus in official or private capacities, and so are able to advise measures which will be acceptable as well as efficient. Recently a commissioner broke his thigh badly, and was sent 16 miles across country, in a bullock cart, to receive nursing and attendance. This commissioner was very much the "biggest man in the place," and if this was the best that could be done for him it is not pleasant to think what the poor must suffer at times. One who gives his experience after four years' residence in Cyprus is greatly in favour of the "travelling dispensary," with a qualified doctor, speaking modern Greek, and believes that in some cases fees would be paid by the villagers, so that although the society would incur the expense of setting up and maintaining the itinerant doctor, there would be some small revenue from the patients themselves.

The society will not thank us for deferring so long any special reference to the new hospital they wish to build in the district of Kyrenia, where there never has been one. Successive commissioners have felt the need, and it is a former commissioner, Captain E. R. Kenyon, R.E., who has been specially active in urging forward this work. An English resident there, Mr. Houston, has promised him an admirable site, to be conveyed when the society is ready to commence building. At present, in this district of Kyrenia, "cases requiring surgical treatment occur, but there is no place where the patient can be kept under the doctor's eye, or where proper nursing can be given. Those who have had much experience of the poor, even in England, know how difficult it is to ensure the simplest instructions being obeyed, the most obvious precautions taken, the most rudimentary sanitation attended to. Only those who know the East can tell how all these difficulties are multiplied in a country where an English cottage would be almost a luxurious dwelling, where scarcely any have an idea of sanitary science, where dirt and insect pests abound." Only £1,500 is needed for what is to be, in the first instance, a small cottage hospital (about ten beds at Kyrenia, and which it is hoped at a later day, when more progress has been made in hospital work at Cyprus, may be enlarged and turned into a convalescent home for the neighbouring district of Nicosia, and possibly also be used by paying patients. The site offered is said to be peculiarly suitable for an institution intended to serve this earlier and later purpose. English ladies and their children can live here all the year round without needing a migration to the hills. There is capital sea-bathing, the water supply is good and abundant, and in the neighbourhood is some of the loveliest scenery in the island. It is hoped to make this hospital, though a small one, a model not only for Cyprus, but also for the neighbouring provinces of Asia Minor, a proposal which may sound ambitious, but surely is a motive to be kept in view; if people see what a hospital can be they will desire to see all their hospitals what they ought to be. This new, small, model institution is to be called the Gordon Hospital. General Gordon was never in Cyprus, but his name is known and honoured there, and it is said his heroic struggle at Khartoum was watched by natives almost as closely as by the few English residents at Kyrenia. This memorial is to be given him, however, because his name will impress on the institution the character it is intended to bear—the character of sympathy for sufferers and of Christian anxiety to help them in body and soul wherever and whenever opportunity shall be given.

